# Impact of epigenetic reprogramming on antitumor immune responses in glioma

**DOI:** 10.1172/JCI163450

**Published:** 2023-01-17

**Authors:** Brandon L. McClellan, Santiago Haase, Felipe J. Nunez, Mahmoud S. Alghamri, Ali A. Dabaja, Pedro R. Lowenstein, Maria G. Castro

**Affiliations:** 1 Department of Neurosurgery and; 2 Department of Cell and Developmental Biology, University of Michigan Medical School, Ann Arbor, Michigan, USA.; 3 Accenture-Argentina, Autonomous City of Buenos Aires (CABA), Argentina.; 4 Boehringer Ingelheim Pharmaceuticals Inc, Ridgefield, Connecticut, USA.; 5 Rogel Cancer Center, University of Michigan, Ann Arbor, Michigan, USA.

## Abstract

Epigenetic remodeling is a molecular hallmark of gliomas, and it has been identified as a key mediator of glioma progression. Epigenetic dysregulation contributes to gliomagenesis, tumor progression, and responses to immunotherapies, as well as determining clinical features. This epigenetic remodeling includes changes in histone modifications, chromatin structure, and DNA methylation, all of which are driven by mutations in genes such as histone 3 genes (*H3C1* and *H3F3A*), isocitrate dehydrogenase 1/2 (*IDH1/2*), α-thalassemia/mental retardation, X-linked (*ATRX*), and additional chromatin remodelers. Although much of the initial research primarily identified how the epigenetic aberrations impacted glioma progression by solely examining the glioma cells, recent studies have aimed at establishing the role of epigenetic alterations in shaping the tumor microenvironment (TME). In this review, we discuss the mechanisms by which these epigenetic phenomena in glioma remodel the TME and how current therapies targeting epigenetic dysregulation affect the glioma immune response and therapeutic outcomes. Understanding the link between epigenetic remodeling and the glioma TME provides insights into the implementation of epigenetic-targeting therapies to improve the antitumor immune response.

## Introduction

Gliomas are the most common type of primary tumors originating in the central nervous system (CNS) (1). They are characterized as highly heterogeneous tumors, both biologically and morphologically. Gliomas are classified according to the WHO classification system in grades 1 to 4, indicating their growth rate and aggressiveness (2). While non-diffuse gliomas can be curable when resected entirely, diffuse gliomas, characterized by their infiltrative nature, are difficult to treat owing to the critical neural structures involved and the limited access of drugs to the brain. Thus complete surgical resection of diffuse gliomas is almost always impossible. For these reasons, recurrence of surgically removed diffuse gliomas is almost unavoidable, and patient outcomes have not improved significantly in recent decades, unlike with other cancers (3). Glioma progression and patient survival depend on the glioma location, the patient’s age, glioma cells’ genetic mutations, and epigenetic dysregulation.

**Figure 1:**
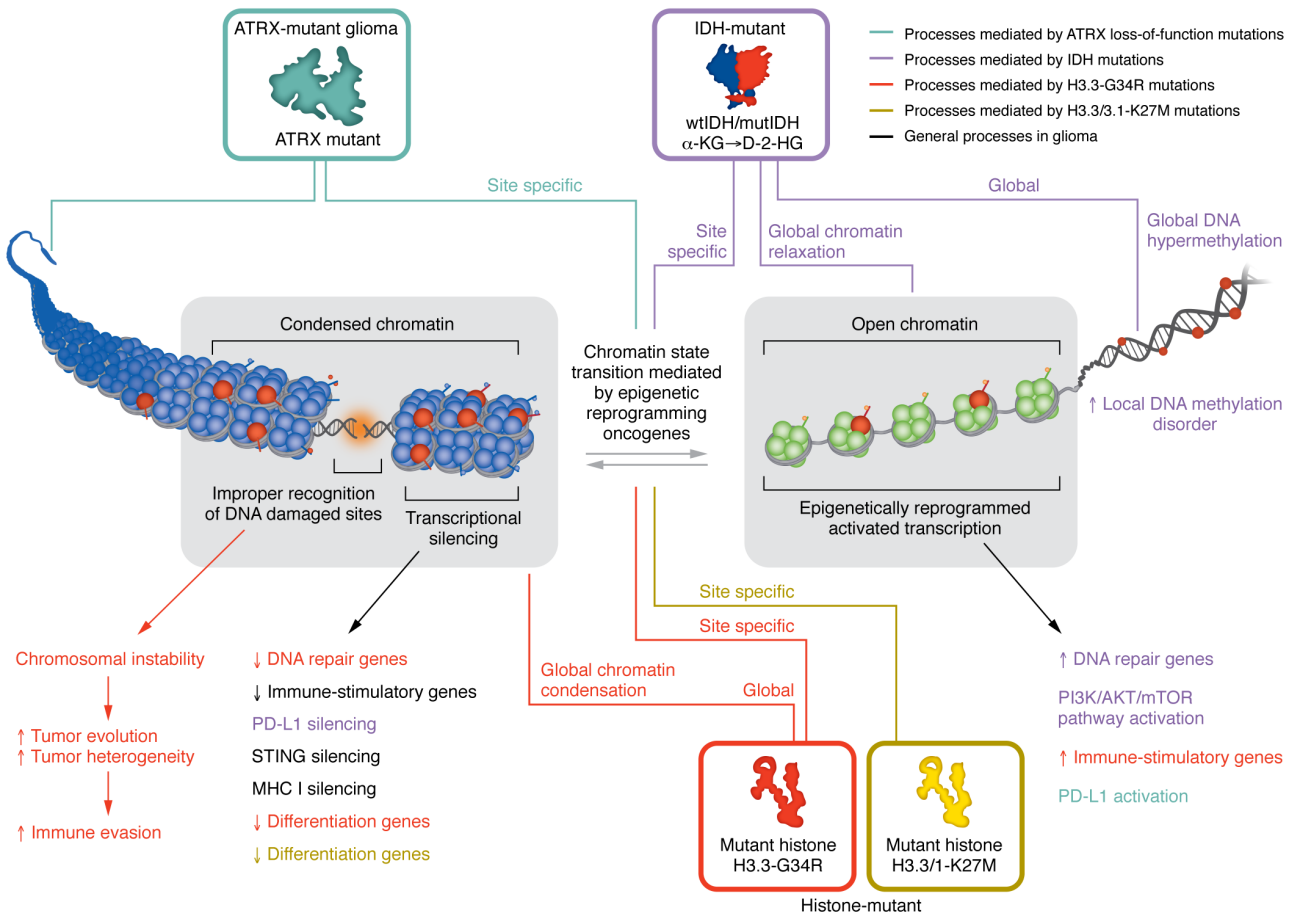
Effects of epigenetic mutations in molecular mechanisms that modulate immune cell activity within the TME. The effects of epigenetic mutations on chromatin are indicated by color-coded arrows. The molecular effects of epigenetic changes that affect the immune TME are indicated below the chromatin.

We first describe the distinct adult and pediatric glioma microenvironments. Then we examine how epigenetic mechanisms modulate the glioma tumor microenvironment (TME) (Figure 1). We also cover the impact of epigenetic-targeting therapies on the immune response. This Review serves to discuss the literature on the impact of epigenetic dysregulation in glioma on several aspects of the glioma microenvironment. The mechanistic aspects of the epigenetic reprogramming have been reviewed in detail in refs. 4–6.

## Adult gliomas

It is well established that gliomas in adults generally differ both clinically and molecularly from pediatric gliomas. Adult gliomas have an incidence rate of about 5.0 per 100,000 people (7). The most prevalent subtype, glioblastoma (GBM), accounts for 57.7% of gliomas and has the most dismal median survival of approximately 15 months, even with standard-of-care treatments (8).

Adult diffuse gliomas comprise 3 types: astrocytoma, IDH-mutant; oligodendroglioma, IDH-mutant and 1p/19q-codeleted; and glioblastoma, IDH-wild-type — which includes the genetic modifiers, the mutation status of isocitrate dehydrogenase (IDH) genes, and codeletion of chromosome arms 1p and 19q (1p/19q codeletion) (2, 9, 10). Other molecular profiles that are characteristically altered in adult gliomas include *ATRX*, *TP53*, TERT promoter, *CDKN2A/B*, *EGFR*, and chromosomes 7 and 10. The most common mutation of IDH is in IDH gene 1, typically at arginine 132 to histidine (IDH1-R132H; mIDH1) (11). mIDH1 astrocytoma patients survive longer than patients with wild-type IDH (wtIDH) (12). In astrocytomas, mIDH occurs early in gliomagenesis and is accompanied by several other mutations, including *ATRX*, *TP53*, and *CDKN2A/B* (13, 14). In oligodendroglioma, the mIDH and 1p/19q codeletion are often accompanied by mutations in TERT promoter, *FUBP1*, and *NOTCH1* (9, 11). Mutations in TERT promoter, *EGFR*, and chromosomes 7 and 10 are associated with GBM (2, 9). Molecular alterations are used in tandem with histopathological characteristics to determine the glioma grade.

## Pediatric high-grade gliomas

Pediatric high-grade gliomas (pHGGs) constitute 8%–12% of all CNS pediatric tumors and have an incidence of approximately 0.85 per 100,000 children (15, 16). pHGG prognosis is dismal, with an overall median survival of approximately 10 to 18 months, and the 2-year survival rate is approximately 32% for hemispheric tumors versus approximately 10% to 22% for midline tumors (17). Despite intense research efforts, currently there are no effective treatment options. pHGG remains the leading cause of cancer-related death in children and adolescents under 19 (15, 18).

Many epigenetic-related mutations are seen more commonly (in some cases almost exclusively) in pediatric gliomas, particularly mutations in histone 3 (H3) genes. These mutations also include chromatin remodelers such as SETD2; α-thalassemia/mental retardation, X-linked (ATRX); and DAXX (5, 17, 19, 20). Midline diffuse gliomas typically harbor the H3.1-K27M or the H3.3-K27M histone mutation, while diffuse hemispheric pHGGs contain the H3.3-G34R/V mutation. ATRX and DAXX are often seen in pHGG and are associated with telomere lengthening (5, 20).

## The unique microenvironment of glioma

### Distinct immune-modulating features of the brain.

Compared with other tumors, gliomas have unique immune characteristics. The anatomical location within the brain as compared with anywhere else in the body mediates a host of distinct features that contribute to alterations in the immune response to the cancer cells. Distinct immune-modulating features of glioma include the blood-brain barrier (BBB) and the presence of neuronal and glial cells.

The BBB contributes to the homeostasis of the CNS via regulation of the influx and efflux of biological substances (21). Although the BBB generally serves to protect the brain from toxic substances in the blood while allowing for the passing of necessary nutrients, this semipermeable layer can also restrict the entry of peripheral leukocytes and therapeutic moieties (22). Inflammation within the brain has been shown to compromise BBB integrity (22, 23), allowing for increased immune cell infiltration, but by the time this occurs, gliomagenesis will likely have already escaped immune surveillance.

Neuronal activity has been shown to promote glioma growth and invasion (24, 25). Signaling molecules released by neurons, such as the synaptic protein neuroligin-3 (NLGN3), can influence glioma progression by activation of the PI3K/mTOR pathway (24, 25). Additionally, a positive-feedback mechanism promotes the expression of neurotransmitters in tumor cells and affects immune cell functions (25–29). The role of epigenetic dysregulation in glioma in altering neuronal behavior remains understudied. Recent independent studies by Venkatesh et al. (30) and Venkataramani et al. (31) demonstrated that glioma cells can establish NLGN3-dependent synaptic connections with neuronal cells in vivo.

Venkatesh et al. showed that oligodendroglial precursor cell–like (OPC-like) glioma cells express synaptic genes (30). Glioma cells can establish two different types of synapses with neurons, one mediated by AMPA (α-amino-3-hydroxy-5-methyl-4-isoxazole propionic acid) receptors (a type of ionotropic glutamate receptor), reminiscent of a neuron-OPC synapse, and the other mediated by potassium currents, reminiscent of a neuron-astrocyte synapse. The authors showed that the expression of GluA2, an AMPA receptor, confers growth advantage to glioma, promoting a decreased survival in vivo, and that coculturing glioma cells with neurons in vitro promotes tumor cell growth, demonstrating the importance of neuron-to-glioma synaptic connections for glioma progression. Venkataramani et al. characterized AMPA receptor–mediated neuron-glioma synapses and demonstrated that these connections promote glioma invasion and proliferation (31). These studies highlight the importance of neurons and synaptic connections in promoting tumor growth and modulating glioma behavior.

The glial cell population consists of three major subtypes: microglia, oligodendrocytes, and astrocytes. Microglia are brain-resident immune cells, and as such, they are discussed in detail in the myeloid cell section. Oligodendrocyte-lineage cells (OLCs), comprising oligodendrocytes and oligodendrocyte precursor cells, can increase the invasive activity, stemness, and chemoresistance of GBM cells by angiopoietin-2, FGF-1, and EGF secretion (32, 33). OLCs have been shown to modulate the immune response via the production of immune-mediating molecules, such as IL-18, IL-6, and CCL2 (34). Astrocytes are the most prevalent glial cells in the CNS (35), which activate and undergo transcriptomic reprogramming to become reactive astrocytes in response to CNS pathologies including glioma (36). In glioma, reactive astrocytes secrete protumor, antiinflammatory cytokines such as IL-6, TGF-β, and VEGF (36, 37).

### Lymphoid cells.

Tumor-infiltrating lymphocytes (TILs) have strong antitumor immune functions and are key targets for immunotherapies. Although both B cells and T cells have been identified in gliomas (38), T cells are the predominant TILs and play a major role in glioma progression; thus the main body of glioma TIL research has been conducted on T cells. Regulatory T cells (Tregs), CD4^+^ T helper (Th) cells, and CD8^+^ T cells have all been shown to infiltrate the glioma TME (39). Tregs strongly suppress the functions of antitumor immune cells and support increased levels of other immunosuppressive cells (40, 41). Th cells, specifically Th1 cells, and CD8^+^ T cells contribute to the antitumor immune response by stimulating increased inflammation and tumor cell killing, respectively. Their antitumor effect is hindered by their low tumor infiltration and the immunosuppressive glioma TME (42, 43). Immune suppression is mediated primarily by glioma cells, myeloid-derived suppressor cells (MDSCs), Tregs, and tumor-associated macrophages and microglia (TAMMs) via the expression of immune checkpoint receptor ligands, such as PD-L1, and the secretion of immunosuppressive cytokines, such as TGF-β and IL-10 (44). These molecules, among others, mediate T cell dysfunction, i.e., anergy and exhaustion (45, 46). Exhausted Th cells and exhausted CD8^+^ T cells have reduced levels of proliferation and reduced effector cytokine production, i.e., IL-2, TNF-α, and IFN-γ (46, 47). T cell exhaustion is accompanied by increased chromatin accessibility and transcription in genes encoding immunosuppressive molecules (*PDCD1*, *CTLA4*, *LAG3*, *ENTPD1*) as well as decreased chromatin accessibility and transcription for genes important for cell differentiation (*IL7R*, *TCF7*, *LEF1*) (48, 49).

### Myeloid cells.

Myeloid cells are the major immune population within the glioma TME, representing about 60% of all infiltrating immune cells (50–52). This population is composed primarily of brain-resident microglia, bone marrow–derived macrophages, and MDSCs (41, 53).

Despite the differences in their developmental origin, microglia and macrophages share several phenotypic properties. Microglia and macrophages can acquire antitumoral or protumoral phenotypes in the TME, but research suggests that the majority of TAMMs in the glioma TME have an immunosuppressive phenotype (54). In addition, TAMM characteristics have been shown to differ based on glioma cells’ mutations (55). For example, antitumoral macrophages are more abundant in wtIDH gliomas compared with mIDH1 gliomas (55).

MDSCs are a heterogeneous subgroup of immature myeloid cells with potent immunosuppressive properties. They promote proliferation and invasion of GBM cells (56). MDSCs are subdivided into polymorphonuclear MDSCs (PMN-MDSCs) and monocytic MDSCs (M-MDSCs), which exert different mechanisms to suppress immunity. PMN-MDSCs are suppressive via reactive oxygen species (ROS), peroxynitrite, arginase 1, and prostaglandin E_2_ production (57). M-MDSCs are suppressive via the expression of immunoregulatory molecules such as PD-L1 and by secreting nitric oxide, IL-10, and TGF-β (57).

## Epigenetic mechanisms associated with TME modulation in glioma

### Transcriptional reprogramming.

**Figure 2:**
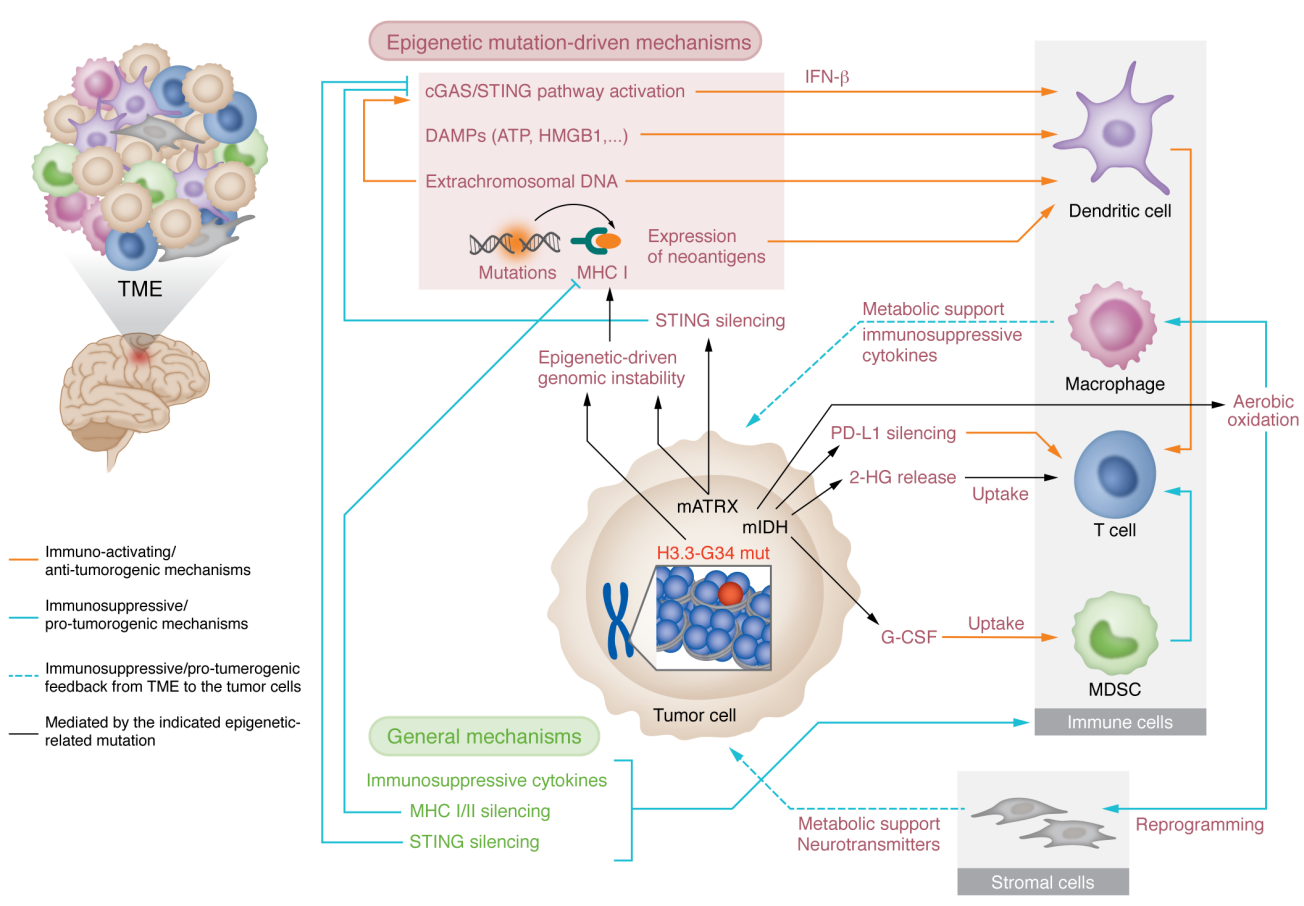
Epigenetic mechanism–mediated interactions between glioma cells and nontumoral cells that shape the TME. Connections are indicated by arrows, and the color of the arrows indicates whether the interactions lead to immunosuppressive/protumoral or immune-activating/antitumoral mechanisms. The start of the black arrows indicates the mutations in the glioma cells that elicit the epigenetic mechanisms.

The most evident mechanism by which epigenetic alterations in glioma manipulate the tumor-infiltrating immune cells is via transcriptional modulation of genes associated with immune activation/suppression (Figure 2). For epigenetically mediated transcriptional modifications on the tumor cells to affect the immune cells, immune cells must sense a tumor-derived signal. The interaction between tumor cells’ surface ligands and immune cells’ receptors is an example of a direct mechanism. It was demonstrated that glioma TME-associated T cells are different from non-infiltrating T cells, and that glioma cells express ligands that can reprogram the T cell toward an exhausted phenotype (58). Our group and others demonstrated that mIDH1 expression mediates the transcriptional silencing of the PD-L1–encoding gene *CD274* via DNA methylation (59, 60). The DNA methylation levels in the regulatory regions of *CD274* decrease when mIDH1 glioma cells are treated with an mIDH1 inhibitor (59). PD-L1 expression by tumor cells supports T cell exhaustion, so mIDH1-harboring gliomas have less PD-L1 to suppress T cell activity. A recent study describes that loss of ATRX, another epigenetic modulator in both adult and pediatric glioma (20), epigenetically induces the expression of PD-L1 and several immunosuppressive cytokines, eliciting tolerogenic mechanisms in ATRX-mutant glioma (61).

**Figure 3:**
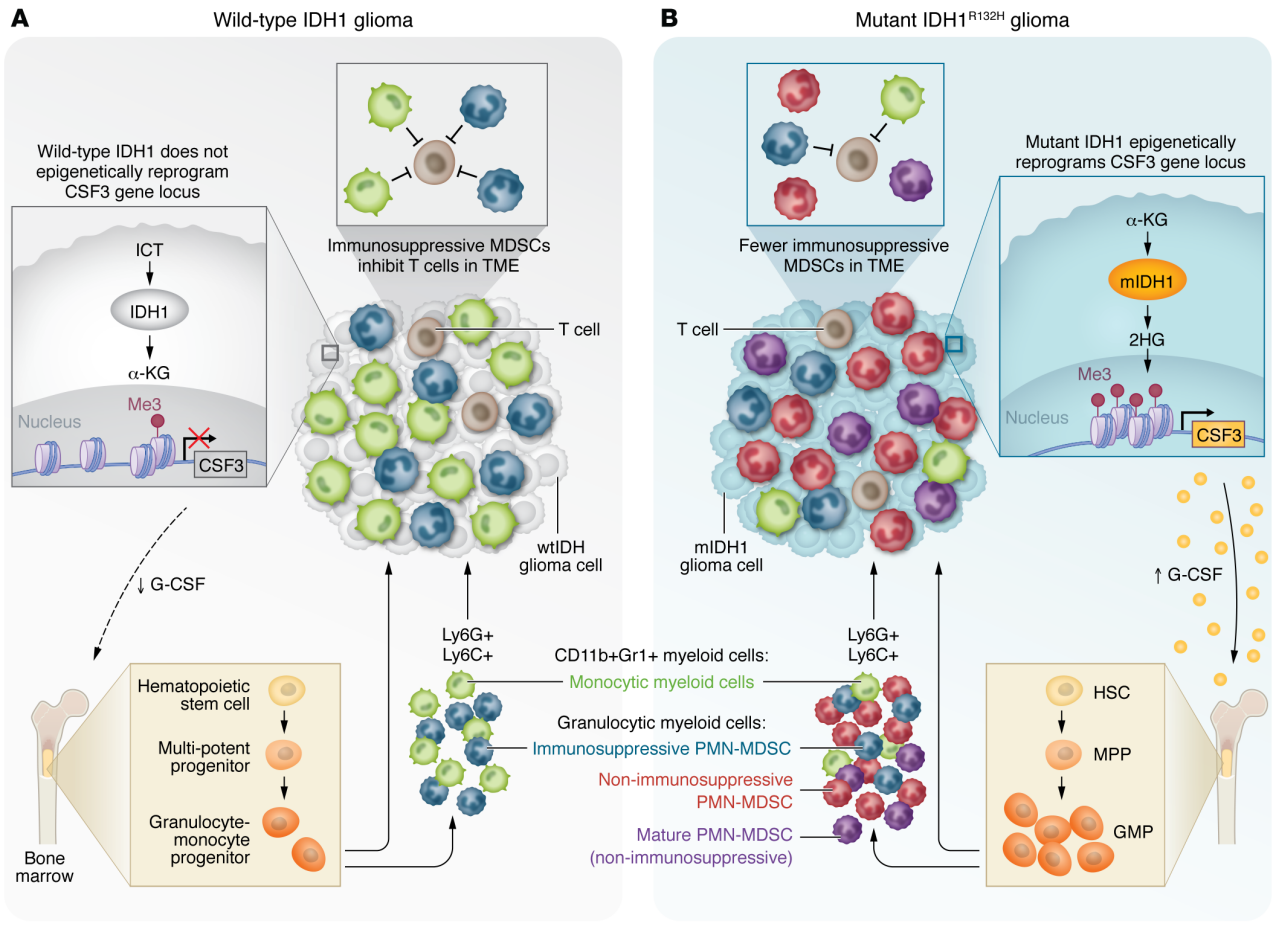
Model of aberrant granulocyte differentiation in mIDH1 tumors. (**A**) In WT IDH1 tumors, tumor cells express low levels of G-CSF and the TME contains a high number of immunosuppressive MDSCs. (**B**) Through epigenetic reprogramming mediated by mIDH1-induced 2-HG accumulation, mIDH1 glioma cells express and secrete G-CSF. Circulating G-CSF has a direct effect on hematopoiesis in the bone marrow and spleen, promoting the expansion, differentiation, and mobilization of granulocytic myeloid cells. As a result, the granulocytes recruited to the TME are mainly neutrophils and preneutrophils, with inhibitory PMN-MDSCs, constituting a smaller fraction of the total granulocytes in the mIDH1 tumor. Figure adapted from Alghamri et al. (63).

Tumor cells can also release cytokines that can be distally sensed by immune cells to activate or suppress the immune response. In particular, glioma cells secrete the immune-modulating cytokines IL-1β, IL-6, TGF-β, and IL-8 (62). Our group demonstrated that granulocyte colony-stimulating factor (G-CSF) expression is epigenetically activated by mIDH1 in glioma stem/progenitor-like cells, mediating reprogramming of myeloid cells within the mIDH1 glioma TME (63). This increased G-CSF prompts the expansion of pre-neutrophils and neutrophils, while reducing the immunosuppressive phenotype of PMN-MDSCs encountered in the mIDH1 TME (Figure 3) (63). Other potential mechanisms by which tumor cells can alter the immune cells distally involve the release of damage-associated molecular patterns (DAMPs). HMGB1 and extracellular ATP, for example, are released by glioma cells and promote inflammation in the TME (64, 65). Tumor cells also produce extracellular vesicles (EVs), which can mediate cell signaling. Glioma-derived exosomes can carry immunosuppressive molecules (66) and were shown to have functional suppressive activity on different immune cell types (66, 67). The role of epigenetic mutations in the regulation of the secretion of EVs and their cargo is an exciting, understudied field that could open opportunities for the development of tailored therapies.

The epigenetic modulation of tumor cells’ differentiation also has consequences for the TME. Glioma epigenetic mutations (in particular, H3 mutations in pediatric glioma and mIDH1 mutations in adults) have been shown to result in stalled differentiation, committing the cells to an undifferentiated, stem-like state. Cancer stem-like cells are less immunogenic, and can evade the immune system through various mechanisms, including major histocompatibility complex (MHC) transcriptional downregulation, induction of quiescence, and other mechanisms that promote immune tolerance. Thus, epigenetically mediated interruption of cell development in early neoplastic stages likely makes the initial tumor development possible by hampering the antitumoral immune response. A recent study shows that glioma stem cells (GSCs) selected to grow in immunocompetent hosts go through a process of epigenetic adaptation, which leads to secretion of immunosuppressive cytokines (68). Moreover, the adapted GSCs show upregulation of IRF8, a cytokine normally restricted to myeloid cells as it controls myeloid lineages and macrophage differentiation (68). This demonstrates that epigenetic mechanisms mediate reprogramming of glioma cells resulting in the modification of immune cells and induction of a pro-tumorigenic TME.

The role of histone mutations as modulators of the immune TME remains understudied. A recent study describes that the transcription factor RACK7 (ZMYND8) has increased affinity for the mutant histone H3.3-G34R (a driver mutation of pHGG). Increased binding of RACK7 to the mutant histone leads to transcriptional repression of selected genes, including *CIITA*, a master regulator of MHC class II expression (69).

Polycomb repressive complex 2 (PCR2) is a family of histone methyltransferases that controls epigenetic silencing, and whose function can be altered in glioma (70). PCR2 proteins’ expression levels were correlated with poor prognosis, and it was suggested that PCR2 chromatin silencing mediates immunosuppression by blocking the expression of immune-stimulatory cytokines in tumor cells (70). A better response to immune checkpoint inhibitors (ICIs) was recently reported for tumors with mutations in PCR2 (70). This opens possibilities to improve the response to immunotherapies by reverting the methylase activity of PCR2 via epigenetic pharmacological modulation (71).

The Notch pathway was shown to be epigenetically modulated in mIDH1 gliomas, through DNA methylation of CpG sites within the delta-like ligand 3 (*DLL3*) gene (72). DLL3 is an inhibitory Notch ligand, and its expression positively correlated with survival in mIDH1 gliomas. mIDH1 gliomas with high expression of DLL3 showed increased immune infiltration, suggesting an association between Notch signaling and immune activity in these tumors (72).

### The role of noncoding RNAs in TME modulation.

In recent decades, noncoding RNAs (ncRNAs) like microRNAs (miRNAs) and long noncoding RNAs (lncRNAs) were shown to play critical roles in glioma, including acting as epigenetic modulators. miRNAs are natural interference RNAs that act via inactivation of mRNA (73). Loss of miRNAs that naturally regulate critical mRNAs can be oncogenic, such as miR-31, which silences CDKN2A/B (74), and miR-34a, which controls EGFR levels (75). The expression of different miRNAs was observed in certain molecular subtypes, indicating that miRNAs can regulate the heterogeneity of glioma by mediating transcriptional subtype transitions (76).

LncRNAs are also involved in glioma biology, progression, and response to therapies (77). The lncRNA HOTAIRM1 acts as an epigenetic regulator by binding to transcription start sites and blocking the access of epigenetic modifiers to regulatory gene regions (78). Similarly, lncSNHG6 and lncRNA ZFAT-AS1 can promote epigenetic silencing by inducing H3K27me3 gene-specific deposition (79, 80).

One characteristic of ncRNAs is that they can function as intercellular signals (81). miRNA and lncRNA secretion by glioma cells can impact TME behavior. For example, lncRNA-ATB secreted by glioma cells can suppress miR-204-3p in astrocytes, which could promote migration of glioma cells (82). Other studies indicate that lncRNAs secreted via exosomes can have a paracrine effect, promoting adaptation to stress/hypoxia conditions and resistance (83, 84). The communication between glioma cells and the TME via ncRNA emerges as an area with great therapeutic potential, although the role of ncRNA in altering the epigenetic landscape of the TME, particularly the immune cells, remains understudied.

### The epigenetic manipulation of metabolism within the TME.

Epigenetic activation of the PI3K/AKT/mTOR pathway is commonly observed in glioma, resulting in selection advantages such as increased metabolism, proliferation, stemness, and invasiveness. This excessive metabolism in glioma cells leads to the switch from oxidative phosphorylation (respiration) to the oxidation of pyruvate to lactate (aerobic glycolysis) (85). This phenomenon, typical of cancer cells, is called the Warburg effect. It was demonstrated that aerobic glycolysis can modulate immune cells’ functions. The release of lactic acid and the resulting hypoxia lead to the induction of an immunosuppressive TME by mechanisms that include increased secretion of TGF-β, inhibition of the monocyte differentiation to dendritic cells by lactic acid, and secretion of pro-tumorigenic cytokines (i.e., IL-23) (86). Additionally, the metabolic alteration of the TME can affect the function of astrocytes to promote the growth of glioma cells (via release of cholesterol) and the recruitment of immunosuppressive macrophages (87).

### d-2-Hydroxyglutarate uptake by immune cells within the TME.

Glioma cells expressing mIDH1 produce increased levels of d-2-hydroxyglutarate (2-HG), resulting in epigenetic reprogramming of the tumor cells (88). The ability of 2-HG to affect the immune TME remains unclear. It has been reported that 2-HG can be internalized by T cells in vitro, and that T cells isolated from mIDH1 acute myeloid leukemia patients have high levels of 2-HG (89). One study found that 2-HG triggers HIF-1α protein destabilization, leading to metabolic skewing, oxidative phosphorylation, increased Treg frequency, and reduced Th17 polarization (89). A recent study found that exposure to 2-HG reduced proliferation of activated T cells, although a study from our group found no effects of 2-HG on T cell proliferation (59). Sodium-dependent dicarboxylate transporter 3 (SLC13A3) and organic anion transporter SLC22A6 were hypothesized to mediate 2-HG internalization by T cells (89, 90). The mechanisms mediating the internalization of 2-HG have yet to be elucidated for the glioma immune TME.

### Genetic instability and immunity.

Besides the transcriptional alterations, epigenetic dysregulation can cause direct effects on the structure of the chromatin. In glioma, histone and ATRX mutations have been associated with genetic instability, which results from abnormal histone mark deposition in these cells (20).

Mutant IDH was shown to epigenetically upregulate the DNA damage response (91). The genetic instability has many consequences, among them the emergence of extrachromosomal DNA in the cytoplasm. This phenomenon activates the cGAS/STING pathway in the tumor cells, leading to the activation of innate immune cells, such as dendritic cells (92). Additionally, epigenetically mediated genetic instability results in the accumulation of chromosomal alterations, promoting the expression of neoantigens arising from mutant proteins (93). These neoantigens can be recognized by adaptive immune cells, leading to immune activation or tolerance, depending on the tolerogenic properties of the TME. Notably, epigenetic regulation of STING (via STING promoter DNA methylation) has been proposed to modulate the immune response in glioma (94). Moreover, STING silencing in glioma can be reversed by DNA methyltransferase inhibition (95). A recent study from our group demonstrated that H3-G34 mutations, present in pHGG, confer genomic instability to these tumors (96). This results in activation of the cGAS/STING pathway, and promotes the activation of the immune system, improving the efficacy of DNA-damaging treatments (96).

### Epigenetic reprogramming in tumor heterogeneity, evasion, and resistance.

Intratumoral heterogeneity in glioma was evidenced by the heterogeneous levels of expression of specific markers in biopsied tissue (97). Recent single-cell high-throughput analyses have helped to uncover the molecular basis of spatial and temporal heterogeneity (98, 99). The intratumoral heterogeneity can be based on genetic differences among the tumor cells, or due to epigenetic differences, which lead to different transcriptional profiles (100). Glioma cells were shown to transition between transcriptional states resembling mesenchymal (MES), astrocytic, neural precursor, and oligodendrocyte precursor lineages (98). A recent study aimed to characterize the interaction of these molecular programs in glioma cells and their interactions with the TME through the integration of spatial transcriptomics and scRNA-Seq from multiple glioma patients (101). The spatial transcriptomics uncovered that diverse molecular regions are recurrent in glioma. One of these niches encompasses tumor areas undergoing hypoxia and composed of MES-like cells. Glioma cells in these regions have increased genomic instability and are proposed as potential sources of adaptive evolution and development of resistance to therapies. Another niche is described as “reactive immune” and is characterized by increased immune infiltration, glioma cells with MES-like phenotype, and expression of immunosuppressive markers. The work also describes that the environment in which the tumor is growing (i.e., species and host age) can determine the molecular phenotypes adopted by glioma cells. This study demonstrated the impact of the intratumoral heterogeneity on the TME and reveals the potential of manipulating the TME to induce changes in the tumor cells.

Epigenetic heterogeneity can impose additional effects on the immune TME, as there can be differences among the immune-stimulating or immunosuppressive activities of different glioma cells according to the location of the cell and the stage of tumor development. As mentioned above, GSCs have immunosuppressive activities, and the epigenetic mutations commonly found in gliomas can affect the stemness of the tumor, as well as the identity of the cells (98). Heterogeneous expression of glioma markers mediated by epigenetic mechanisms has been mentioned as one factor limiting the success of chimeric antigen receptor (CAR) T cell therapies (102). Local DNA methylation disorder is another common dysregulation in glioma cells, particularly in those with mutations affecting epigenetic machinery (99, 103, 104). In response to stresses (i.e., hypoxia and irradiation), DNA methylation disorder increases, and it was speculated that this can provide a mechanism by which the plasticity of glioma cells increases to adapt to stressors.

Heterogeneity also plays a main role in the adaptation of gliomas to treatments, as it contributes to the generation of a larger population of phenotypes from which resistant cells will emerge. After the treatment, a fraction of the cells survive (i.e., resistant cells), leading to a transient reduction of tumor heterogeneity. Thus, a strategy recently proposed to treat glioma aims to target this window when heterogeneity is reduced, as the tumor cells have reduced plasticity to adapt to a second treatment.

## Immune effects of therapies targeting epigenetic remodeling

The glioma epigenetic landscape revealed several epigenetic alterations that are mechanistically associated with tumor behavior (105, 106). Alterations in DNA methylation, histone methylation/acetylation, and IDH mutation status are frequent in gliomas and susceptible to targeting using epigenetic therapies. Strategies to target the glioma epigenome have been demonstrated to be effective in controlling tumor growth and represent a valuable alternative owing to the ability to reverse the epigenetic dysregulation that supports brain tumors (107). In fact, several DNA methyltransferase inhibitors, histone deacetylase inhibitors, PRC2-EZH2 methylase inhibitors, and mIDH1 inhibitors are currently being evaluated in clinical trials (108). Also, epigenetic processes in glioma cells can mediate the immune response. Thus, therapies targeting epigenetic mechanisms can be used to boost antitumor immunity (11).

### DNA methyltransferase inhibition.

DNA methyltransferases (DNMTs) are enzymes that catalyze DNA methylation and regulate biological functions by modulating gene transcription (109). DNMTs catalyze the formation of 5-methylcytosine from cytosines in DNA CpG islands and ultimately suppress gene expression (109). Atypical DNMT functionality is often associated with tumor development via mechanisms leading to hypermethylation of tumor suppressor genes and increased genomic instability (109). DNMT inhibitors (DNMTis) can restore tumor suppressors’ activity by blocking DNA methylation, thereby reducing tumor cell proliferation and inducing apoptosis (110). Because DNA methylation processes are crucial for immune cell lineage progression and functionality (111), DNMTis can play a direct role in modulating antitumor immunity, yet the direct effects of DNMTis on immune cells remain unestablished. However, DNMTis can promote tumor-specific CD8^+^ T cell activation by upregulating MHC class I antigen presentation by glioma cells (112). Since T cell exhaustion is characterized by altered gene expression linked with alterations in DNA methylation by DNMTs (113), DNMTis may also promote antitumor immunity by preventing T cell exhaustion. Deleting a DNMT enzyme, DNMT3A, in CAR T cells was shown to prevent exhaustion and promote antitumor immunity (114). Therapies that include DNMTis, such as azacytidine and decitabine, are still in the early phases for safety and tolerability testing (Table 1). The ability of DNMTis to revert the chromatin structure, which is characteristic of T cell exhaustion, and enhance the efficacy of anti–PD-1 antibodies supports their use in combination with ICIs (115).

### Histone deacetylase inhibition.

Some common epigenetic changes in tumor cells are related to dysregulated histone mark deposition on regulatory regions in oncogenes and tumor suppressor genes (116). Aberrations in histone deacetylase (HDAC) expression in tumor cells cause altered cell cycle progression and can drive tumor development (116). Studies have linked HDAC expression with glioma grade (117, 118), i.e., lower expression of HDACs class II and IV was found in GBM compared with low-grade astrocytomas (117). Interestingly, HDAC1 was significantly overexpressed in several gliomas and is associated with dismal overall survival (118). While HDAC inhibitors (HDACis) have traditionally been investigated for their ability to target the aberrant epigenetic characteristics of tumor cells, they also induce changes in the antitumor immune response. HDACis enhance T cell chemokine expression, augment responses to PD-1–targeting immunotherapy, and upregulate PD-L1 and HLA-DR on tumor cells (119). This suggests that the combination of HDACis with ICIs could be a valuable therapeutic strategy for glioma patients. The HDACis vorinostat, belinostat, and fimepinostat are being evaluated in clinical trials for both adult and pediatric gliomas (Table 1). However, vorinostat exhibited toxicity and low effectiveness (120). This could be due to its poor BBB penetration (120). Combining HDACis with other therapies and improving HDACi BBB permeability may increase efficacy.

### Mutant IDH1 inhibition.

IDH1 mutation catalyzes the production of 2-HG, which is a competitive inhibitor of α-ketoglutarate–dependent dioxygenases including Jumonji-C domain–containing histone demethylases and the DNA demethylase TET2, generating a hypermethylated phenotype (121). Blocking 2-HG production can reverse DNA hypermethylation and promote differentiation in mIDH1 glioma cells (122). Several inhibitors of mIDH1 were shown to be effective in vitro and in vivo (59, 122, 123). The combination of current standard-of-care therapy (radiation and temozolomide) with mIDH1 inhibitor and PD-L1–blocking ICI increased tumor regression of mIDH1 glioma–bearing mice, decreased T cell exhaustion, and favored the generation of memory CD8^+^ T cells (59). Currently there are several clinical trials testing mIDH1 inhibitors (AG-120, AG-881, DS-1001b, BAY1436032) for glioma treatment, but these are still ongoing and in early phases of determining the safety profiles and ability to decrease 2-HG accumulation (Table 1) (124).

### EZH2 inhibition.

Enhancer of zeste homolog 2 (EZH2) is a histone methyltransferase subunit of PCR2 that is altered in gliomas. This alteration leads to both gain- and loss-of-function activities (125). The aberrant expression of EZH2 impacts gene expression by binding to promoter regions and affecting methylation status, playing an oncogenic role in glioma (126). EZH2 controls the coordinated inactivation of several tumor suppressor genes, thereby promoting cancer growth, invasion, and drug resistance (126). EZH2 inhibitors (EZH2is) enhance p16 tumor suppressor gene expression, affecting glioma progression (125). Importantly, several studies suggest that EZH2 is a critical driver of immune response modulation by cancer cells, mediating immune evasion by downregulation of genes involved in immune activation, upregulation of immune checkpoints, and generation of an immunosuppressive TME (127). In addition, EZH2is increase T cell tumor infiltration, decrease tumor growth, and improve the therapeutic efficacy of ICIs in preclinical tumor models (128, 129). Several studies show that EZH2is decrease proliferation of glioma cells by halting cell cycle progression and altering the proinflammatory response (127, 130). In midline diffuse glioma, repression of EZH2 in microglia induces an antitumor phenotype resulting in decreased cancer cell invasion capability, increased phagocytosis by microglia, and tumor cell death (131). These studies suggest that EZH2is could improve glioma immunotherapy efficacy.

### Challenges of epigenetic therapies.

As implied throughout this Review, epigenetic therapies have potential due to their antitumoral properties, but there are still challenges that must be overcome before epigenetic therapies become widely used. The main challenges are the induction of off-target effects, inability of drugs to cross the BBB, inability of drugs to penetrate into the tumor, and lack of efficacy given the heterogeneous nature of gliomas. Notably, the latter two problems are not specific to epigenetic therapies. Rather, they are common challenges among antiglioma therapies, including immunotherapies. Perhaps the largest hurdle more specific to epigenetic therapies is the chance for off-target effects. Epigenetic therapies target mechanisms either directly, e.g., mIDH1 inhibitors, or by broad epigenome reprogramming, e.g., HDACis and DNMTis. The direct mechanism is ideal for targeting specific mutations that contribute to alterations in epigenetic pathways. Contrarily, the broad epigenome-reprogramming therapies target general epigenetic mechanisms, which play a role in normal cellular processes in cancerous and non-cancerous cells alike. It is these broad epigenome-altering therapies that may result in more side effects. To limit the side effects of epigenetic therapies, it would be beneficial to reduce the volume of these drugs by optimizing the therapeutic window and combining them with other, more targeted treatments such as immunotherapies. Reduction of these off-target effects may also be accomplished through the use of targeted drug delivery systems.

## Discussion

The ability of glioma cells to promote an immunosuppressive environment allows them to circumvent immune rejection. Glioma cells are naturally selected to exhibit molecular hallmarks that allow them to be ignored by the immune system. As gliomas are characterized by the occurrence of mutations that disrupt the epigenetic regulatory mechanisms, here we have discussed how epigenetic dysregulation provides an avenue for tumor evolution to occur. We have also discussed how the molecular intervention of these epigenetically driven mechanisms can be exploited to activate antitumoral immune activity.

An exciting recently uncovered field of study aims at exploiting signals emitted by DNA damage and genomic instability to promote an immune response (96). DNA-damaging therapies, such as radiation therapy and temozolomide, have historically been used for the treatment of gliomas, but these treatments are unable to overcome the immunosuppressive TME. This indicates that the mechanisms that connect DNA damage and innate immunity need to be further stimulated to elicit an effective immune response. In this sense, the direct stimulation of the cGAS/STING pathway via agonist small compounds emerges as an attractive therapeutic strategy (96, 132). *STING* epigenetic silencing can be an immune evasion mechanism in gliomas, so the epigenetic activation of *STING* expression is another possible therapeutic target. Likewise, inducing the expression of MHC class I and class II proteins, which are commonly silenced by epigenetic alterations in glioma, can promote antigen presentation and stimulate the immune system (133). In this sense, recent advances in the development of CRISPR/Cas9–based site-specific epigenetic editing systems allow gene-specific epigenetic therapies to be envisaged.

The epigenetically mediated chromosomal instability in some glioma subtypes is also a relevant mechanism that might allow for the selection of cells that have gained or lost genes that promote immune evasion or resistance. At the same time, the mutational burden imposed by evolutionary selection increases the amount of neoantigens expressed by tumor cells, providing opportunities to elicit adaptive antitumoral immune responses. The immunosuppressive TME does not normally allow for the neoantigens to prime an antitumor response, as the T cells become tolerized because of the lack of necessary costimulatory signals.

Intratumoral TME heterogeneity has been described in detail, in relation to tumor cells, stromal cells, and the immune compartment (98). A fraction of intratumoral heterogeneity cannot be explained by genetic differences among tumor cells, but rather has an epigenetic origin. Dysregulations observed in both adult and pediatric gliomas have been demonstrated to increase epigenetic plasticity and promote identity ambiguity in the tumor cells. Histone mutations lead to developmental stalling that promotes stem-like states that are more apt to induce a tolerogenic TME. Epigenetic intervention to promote differentiation of these stem cell populations within the tumor could reprogram the immune TME toward more antitumoral activity.

Understanding the mechanisms by which tumor cells induce an immunosuppressive TME should provide insights into how to manipulate the immune cells directly. We describe how aerobic oxidation (Warburg effect) in the tumor cells affects the immune cells, reprogramming the immune compartment to a tolerogenic environment. The blockade of pathways that sense metabolic stress in immune cells can be envisaged as a target to avoid this phenomenon. Likewise, the delivery of cytokines into the TME can reverse the immunosuppressive milieu mediated by tumor cells. Notably, diverse glioma mechanisms lead to the establishment of an immunosuppressive TME at the neoplastic stage of tumor development. At the time of treatment, the immunosuppressive TME is already established, and reverting it imposes a complex challenge. In summary, our improved understanding of the critical role that epigenetic processes play in shaping the TME and the immune response to glioma, together with the development of pharmacological agents to manipulate these processes, presents a new promising era of therapies that brings hope for the treatment of otherwise lethal tumors.

## Figures and Tables

**Table 1:**
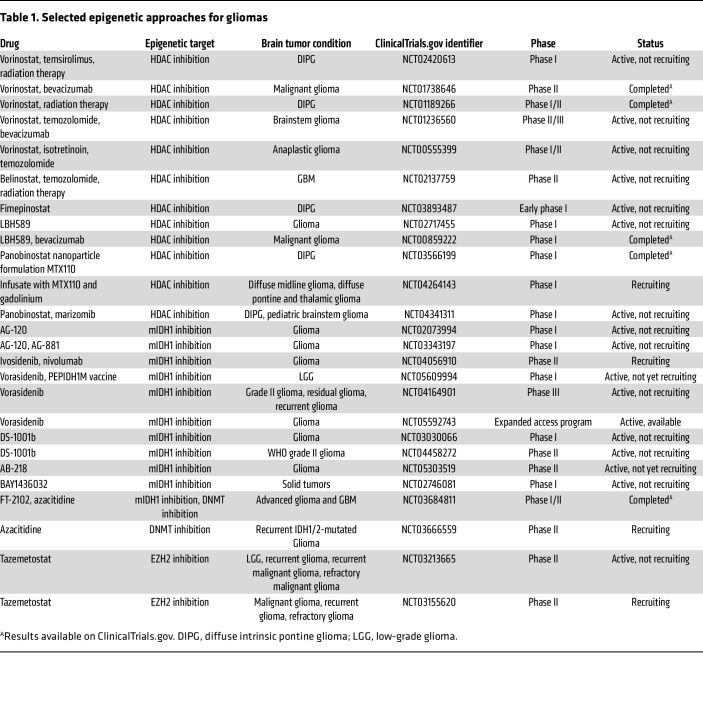
Selected epigenetic approaches for gliomas

## Version 1 01/17/2023

Electronic publication
